# CMV Ileitis: To Treat or Not to Treat? Implications of Initiating Biologic Therapy for Concurrent Crohn's Disease

**DOI:** 10.1155/2019/4513795

**Published:** 2019-06-20

**Authors:** Kendra T. Stilwell, Jason Estes, Maria T. Kurtz, James M. Francis, David T. Lynch, Anish A. Patel

**Affiliations:** ^1^Department of Internal Medicine, Brooke Army Medical Center, San Antonio, TX, USA; ^2^Department of Gastroenterology and Hepatology, Brooke Army Medical Center, San Antonio, TX, USA; ^3^Department of Pathology, Brooke Army Medical Center, San Antonio, TX, USA

## Abstract

Cytomegalovirus (CMV) enteritis is traditionally thought to be a self-limited infection in immunocompetent individuals. Consequently, current guidelines recommend against treating nonimmunocompromised patients with antiviral therapy. Conversely, recent data suggests that spontaneous resolution occurs less frequently than previously believed; furthermore, mortality rate in immunocompetent individuals is similar to that of the immunosuppressed. We present a case of a 43-year-old male who was simultaneously diagnosed with CMV ileitis and Crohn's Disease. When discovered concomitantly, there is no guidance in the current medical literature regarding the benefit of antiviral treatment of the CMV infection prior to initiating biologic therapy versus the risks of withholding treatment, as is currently recommended for nonimmunosuppressed individuals.

## 1. Introduction

Cytomegalovirus (CMV) is in the* Herpesviridae* family and often remains latent after primary infection. The virus later reactivates, often triggered by immunosuppression, either endogenous such as acquired immunodeficiency syndrome (AIDS) or iatrogenic, such as patients undergoing treatment for inflammatory bowel disease (IBD) [1]. Local reactivation has also been linked to onset of inflammatory conditions such as inflammatory bowel disease, specifically ulcerative colitis, even prior to initiation of immunosuppressing therapy. Clinically, distinguishing between primary infection and reactivation of previously acquired latent infection is difficult to establish and likely does not have significant implications for management.

In immunocompetent hosts, primary infection is either asymptomatic or presents as an undifferentiated mononucleosis-like syndrome. CMV infection of the gastrointestinal tract most commonly involves the rectum or esophagus, only rarely affecting the small bowel. In immunocompetent patients, CMV enteritis is traditionally thought to be a self-limited infection, thus current guidelines recommend supportive management only, without antiviral therapy. However, guidance is lacking regarding whether treatment of active CMV infection is recommended prior to initiation of biologic therapy. The only guidelines addressing diagnosis and treatment of CMV prior to immunosuppressing therapy are chemotherapy guidelines for cancer patients.

## 2. Case

A 43-year-old African American male was referred to the Gastroenterology clinic for a 12-month history of alternating diarrhea/constipation, intermittent sharp rectal pain, as well as a 6-week history of pencil-thin stool and staining with defecation. He denied any other constitutional symptoms such as fever, chills, weight loss, or fatigue. A diagnostic colonoscopy was attempted, but limited due to a severe anal stricture.

**Figure 1:**
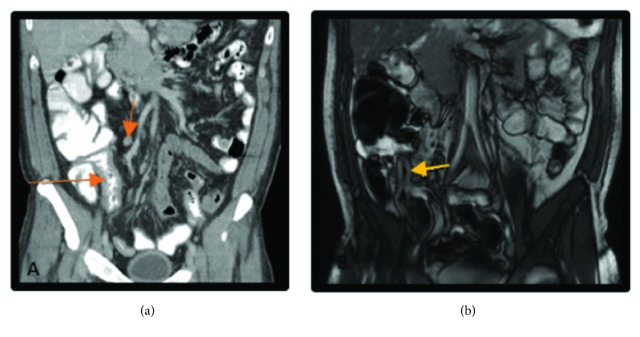
CT, MR Imaging. (a) Coronal CT, irregular wall thickening of the TI (long orange arrow), and mild RLQ lymphadenopathy (short orange arrow). (b) MRE, wall thickening of TI (yellow arrow).

**Figure 2:**
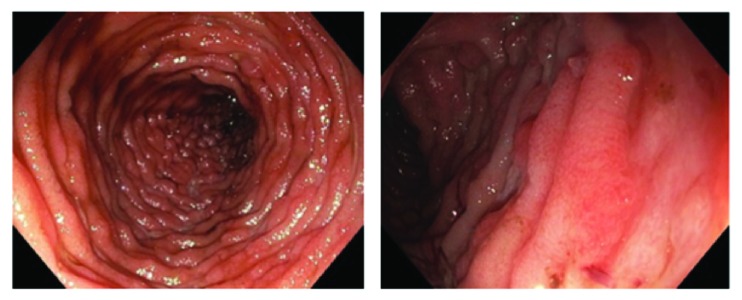
Colonoscopy images. Extensive involvement of the terminal ileum with circumferential ulcerations and inflammation (left). Detailed view of erosions and inflammation seen throughout the ileum (right).

**Figure 3:**
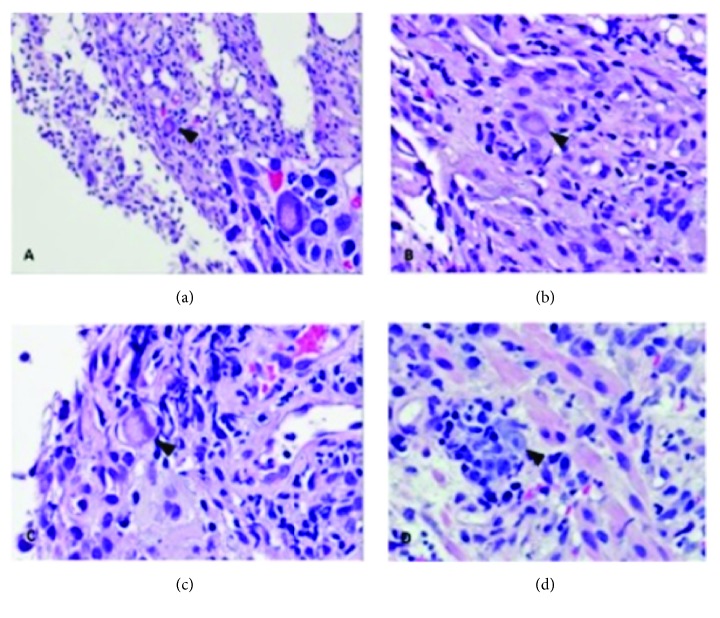
Biopsies from terminal ileum. (a) 200x and 500x (inset) H&E stained appearance of CMV-infected cells. (b) 400x H&E stain showing a cell with CMV cytopathic effect. (c) 500x H&E stain showing a cell with another variation of CMV cytopathic effect. (d) 500x H&E stain with another example of CMV cytopathic effect. The nucleus contains a viral inclusion.

Computed Tomography (CT) and subsequent Magnetic Resonance Imaging (MRI) of the abdomen/pelvis showed a diffusely distended colon and dilated ileum concerning for ileus or enterocolitis, likely infectious or inflammatory in etiology (Figure 1). Rectal exam under anesthesia was notable for a functional narrowing of the anus and two large ulcers at the posterior anal canal. Anal biopsies revealed granuloma formation and positive immunohistochemical staining for CMV. Ileocolonoscopy performed under sedation and monitored anesthesia care demonstrated extensive circumferential ulcerations and inflammation of the terminal ileum (TI) with endoscopically normal colon (Figure 2). Nearly all TI biopsies were positive for scattered CMV-infected cells in a background of diffuse histopathologic effect and ulceration (Figure 3). Unfortunately, a plasma CMV viral load was not checked during his admission as it was unlikely to change management at time; however it would have been useful to demonstrate extent of disease burden and response to treatment.

During his hospitalization, the patient had persistent, frequent bloody bowel movements associated with significant abdominal pain. On hospital day 2, the patient became septic, manifested by fever, tachycardia, tachypnea, leukocytosis of 20.82 x10^3^_,_ and an anion-gap metabolic acidosis. He was initially treated with empiric broad-spectrum antibiotics and fluid resuscitation. Blood cultures were drawn and later grew* Pseudomonas aeruginosa *and* Eggerthella lenta*, both enteric pathogens likely translocated from the bowel due to severe enterocolitis. A thorough workup for underlying immunodeficiency, including human immunodeficiency virus (HIV), quantitative immunoglobulins, flow cytometry for cluster of differentiation 4^+^ (CD4^+^), CD3^+^, CD8^+^, CD19^+^, and CD 56^+^ counts, was unremarkable.

Given the severity of illness, we had significant concerns about initiating immunosuppressive therapy for his Crohn's Disease in the setting active CMV infection. Given the unremarkable workup for underlying immunodeficiency, the infectious disease team recommended against antiviral therapy, in accordance with current guidelines [2]. However, these guidelines do not take into account the risk of beginning immunosuppressive therapy in the setting of severe, active CMV infection. Given the paucity of data available in the medical literature and the significant risk associated with iatrogenic immunodeficiency, the patient was started on valganciclovir 900mg by mouth twice daily for 21 days in addition to levofloxacin and metronidazole for his bacteremia. The patient clinically improved with initial broad-spectrum antibiotic treatment for his bacteremia and continued to experience improved gastrointestinal symptoms after initiation of antiviral therapy. Repeat ileocolonoscopy after completion of the 21-day curse of valganciclovir demonstrated marked improvement of ileitis. Biopsies of the TI, colon, and rectum were negative for continued CMV infection. The patient was then initiated on methotrexate and infliximab therapy for the treatment of newly diagnosed Crohn's Disease with good response on further outpatient follow-up.

## 3. Discussion

CMV is a common latent infection that can reactivate during times of severe immunosuppression, such as in acquired immunodeficiency syndrome, transplant patients or patients undergoing immunosuppressing chemotherapy. When the GI tract is involved, CMV classically affects the esophagus or colon. CMV ileitis is rare, especially in young, immunocompetent individuals. In patients with known IBD, baseline elevation of inflammatory cytokines, such as tumor necrosis factor-*α* (TNF-*α*) and interferon-*γ* (IFN-*γ*), promotes reactivation of latent CMV and can subsequently lead to IBD exacerbation [3]. Moreover, in this population, CMV enterocolitis can lead to severe complications, including toxic megacolon, fistula formation, perforation, and peritonitis. CMV-specific immunoglobulin (Ig) G seropositivity in the United States is estimated at 40-90%, increasing with age. A study demonstrated a 36% seropositivity in children from 6 to 11 years old, increasing to a 91% seropositivity in adults over 80 years old [4]. Unfortunately, levels of these titers do not reliably predict the risk of CMV reactivation and therefore cannot guide decision-making regarding antiviral therapy [5].

CMV infection in immunosuppressed individuals is associated with severe sepsis and high mortality rates. Though CMV infection is believed to be self-limited in immunocompetent hosts, prior studies note that spontaneous resolution of CMV colitis occurred in only 50% of immunocompetent individuals without other comorbidities [6]. Recent data from a 2017 retrospective cohort study of 69 patients suggests CMV enterocolitis in hosts with intact immune systems may carry a 26% mortality rate, equal to their immunosuppressed counterparts [7]. The scope of this study is limited due to the study size and the inability to quantify the attributable mortality to CMV infection versus other confounding factors as >50% of patients in the study presented with sepsis. However, it is important to note that severe CMV infection is common in immunocompetent patients, despite current guidelines reassurance of self-limited disease in this population. Additionally, the gastrointestinal tract was the site of infection in one-third of immunocompetent adults who developed severe CMV infection, defined as any patient requiring hospitalization or deemed life-threatening [8].

Biologic therapy commonly used to treat moderate to severe Crohn's Disease includes TNF-*α* inhibitors, such as infliximab, adalimumab, and certolizumab pegol. TNF-*α* is produced by activated macrophages and T-cells. It is important for macrophage activation, neutrophil chemotaxis, granuloma formation, and maintenance of granuloma structure. The American College of Gastroenterology recommends anti-TNF agents be used in combination with immunomodulatory therapy (such as thiopurines) in moderate to severe Crohn's Disease, as combination therapy is more effective than either treatment class alone in patients naïve to such agents [9].

Although anti-TNF agents offer a more targeted strategy than traditional nonspecific immunosuppressive agents, such as corticosteroids, methotrexate, and azathioprine, multiple adverse effects, including risk of serious infections, have been reported. Consensus amongst experts recommends screening for certain infections prior to initiation of anti-TNF therapies, including tuberculosis, hepatitis B, and hepatitis C. Furthermore, the American College of Rheumatology recommends against initiation of anti-TNF agents if active or recent bacterial, herpes zoster, or invasive fungal infection or nonhealing skin ulcer [10]. None of these guidelines address other active viral infections, such as CMV.

Current medical literature is limited regarding treatment of active CMV infection prior to initiation of biologic therapy. Existing guidelines only pertain to treatment of CMV prior to chemotherapy in cancer patients. National Comprehensive Cancer Network (NCCN) guidelines recommend screening for and treatment of CMV infection prior to immunosuppressive treatments due to the risk of disseminated CMV after starting chemotherapy [11]. More research is needed to determine the best strategy for managing patients with active CMV infection prior to starting biologic therapy for IBD. Physicians should continue to report cases of CMV ileitis in the setting of IBD to increase awareness and facilitate discussion regarding management. Furthermore, physicians should suspect Crohn's Disease in a healthy person presenting with CMV ileitis. Disseminated CMV can be a severe, morbid complication of biologic therapy in the setting of CMV infection, and thus, antiviral therapy should be considered prior to IBD treatment.
